# Identification and Verification of Necroptosis‐Related Genes in Patients With Sepsis by Bioinformatic Analysis and Molecular Experiments

**DOI:** 10.1111/jcmm.70582

**Published:** 2025-05-03

**Authors:** Hayoung Choi, Jin Young Lee, Hongseok Yoo, Kyeongman Jeon

**Affiliations:** ^1^ Division of Pulmonary, Allergy, and Critical Care Medicine, Department of Internal Medicine Hallym University Kangnam Sacred Heart Hospital, Hallym University College of Medicine Seoul Republic of Korea; ^2^ Division of Pulmonary and Critical Care Medicine, Department of Medicine Samsung Medical Center, Sungkyunkwan University School of Medicine Seoul Republic of Korea; ^3^ Department of Health Science Technology SAIHST, Sungkyunkwan University Seoul Republic of Korea

**Keywords:** biomarkers, inflammation, necroptosis, RNA‐seq, sepsis

## Abstract

Although necroptosis is an emerging mechanism of multiple organ dysfunction in sepsis, data on the mechanistic link between necroptosis and sepsis are scarce. Bioinformatic analysis was performed to compare the gene profiles between the sepsis (*n* = 133) and healthy control (*n* = 12) groups and identify necroptosis‐related differentially expressed genes (DEGs). The identified necroptosis‐related DEGs were verified by three‐step molecular experiments: (1) quantitative real‐time PCR and enzyme‐linked immunosorbent assay; (2) cell culture, transfection and Western blotting; and (3) cytokine array with apoptosis inhibition. Additionally, receiver‐operating characteristic curve analyses were performed to evaluate the performance of the corresponding proteins to the necroptosis‐related DEGs in diagnosing sepsis and in predicting in‐hospital mortality of patients with sepsis. Eight necroptosis‐related DEGs, including five upregulated (PYGL, TNF, CYLD, FADD and TLR3) and three downregulated (TP53, FASLG and NLRP6) DEGs, were identified. Moreover, the levels of the corresponding proteins to necroptosis‐related DEGs showed excellent or considerable accuracy in diagnosing sepsis and in predicting the mortality of sepsis patients. In cell culture media transfected with plasma from the sepsis and control groups, Western blotting revealed that the levels of the corresponding proteins were increased in the upregulated DEGs and decreased in the downregulated DEGs. The cytokine array revealed cytokines in cell culture media transfected with plasma from patients with sepsis while preventing apoptosis by inhibiting the caspase‐8 activity, wherein the transfected cells potentially underwent necroptosis. Eight necroptosis‐related DEGs were identified in patients with sepsis by bioinformatic analysis and verified by molecular experiments, implying that necroptosis may be a key mechanism of sepsis.

**Abbreviations**
AUC: area under the curve
CI: confidence interval
CYLD: Cylindromatosis Lysine 63 deubiquitinase
DAMPs: damage‐associated molecular patterns
DEGs: differentially expressed genes
ELISA: enzyme‐linked immunosorbent assay
FADD: Fas associated via death domain
FasL: Fas ligand
GO: Gene Ontology
HMGB1: high‐mobility group box‐1
IL: interleukin
IQR: interquartile ranges
KEGG: Kyoto Encyclopedia of Genes and Genomes
MLKL: mixed‐lineage kinase domain‐like
NLRP6: NOD‐like receptor family pyrin domain containing 6
PYGL: glycogen phosphorylase L
qPCR: quantitative real‐time polymerase chain reaction
RIPK3: receptor‐interacting kinase 3
ROC: receiver‐operating characteristic
SMC‐RoCI: Samsung Medical Center Registry of Critical Illness
TNF: tumour necrosis factor

## 1 Background

Necroptosis is a genetically regulated form of necrotic cell death that has emerged as an important mechanism of multiple organ dysfunction in sepsis [1, 2]. This is supported by the findings of a previous study revealing that the plasma level of the Fas ligand (FasL), a trigger of necroptosis, was associated with the severity of sepsis and was predictive of mortality [3]. Furthermore, the plasma levels of necroptosis mediators, including high‐mobility group box‐1 (HMGB1), receptor‐interacting kinase 3 (RIPK3) and mixed‐lineage kinase domain‐like (MLKL) proteins, were well correlated with the severity and mortality in sepsis [4]. Although previous clinical studies have reported the associations between necroptosis‐related markers and sepsis, they are limited in elucidating the mechanistic link between necroptosis and sepsis.

Bioinformatic approaches integrating computational and life sciences via data mining, pathway and statistical analyses and visual processing have been widely used to investigate the disease on the molecular level [5, 6, 7, 8]; thus, these methods can be a potential solution to illuminate the mechanistic link between necroptosis and sepsis. Our previous study performed a bioinformatic analysis to compare the gene expression profiles between the sepsis and control groups and revealed some key genes that potentially serve as biomarkers for diagnosing sepsis and predicting the outcomes of the sepsis group [8]. As an expansion of the prior study, the present study aimed to identify necroptosis‐related differentially expressed genes (DEGs) in the sepsis group and to compare the data with that of the control group by bioinformatic analysis and to verify the identified DEGs and corresponding proteins by various experimental methods.

## 2 Methods

### 2.1 Study Design and Population

The Samsung Medical Center Registry of Critical Illness (SMC‐RoCI) was used when enrolling the patients with sepsis to our study. The SMC‐RoCI is a prospective observational cohort study conducted at Samsung Medical Center (a 1989‐bed, university‐affiliated, tertiary referral hospital in Seoul, Republic of Korea) [1, 4, 8]. As the third edition of the International Consensus Definitions for Sepsis and Septic Shock (Sepsis‐3) was used to identify patients with sepsis [9], the patients enrolled before the release of this new definition were reclassified according to the Sepsis‐3 scheme. In‐hospital mortality was used to determine the dead and survived groups in patients with sepsis. For the control group, 12 healthy volunteers aged ≥ 19 years donated blood specimens for research purposes and were enrolled in this study.

Written informed consent was obtained from all study participants or their legally authorised representatives before enrolment. This study was conducted according to the guidelines stipulated in the Declaration of Helsinki, and the patient registration and blood sample collection were approved by the institutional review board of the Samsung Medical Center (application no. 2013‐12‐033).

### 2.2 Blood Sample Collection and RNA Isolation

Regarding blood samples, 19 mL of whole blood was collected and stored into ethylenediaminetetraacetic acid tubes within 48 h of enrolment in the SMC‐ROCI. The samples were centrifuged at 480 × g (Eppendorf Centrifuge 5810 No. 0012529‐rotor A‐4‐81) for 10 min at 4°C within 4 h of collection. Several plasma aliquots from each study participant were then isolated and stored at −80°C for further analysis.

Whole blood (2 mL) was also collected for RNA isolation using BD PAXgene blood RNA tubes (BD, cat. no. 762165). Total RNA was isolated from the whole blood using the TRIzol reagent (Invitrogen, Carlsbad, CA, USA) following the manufacturer's protocol [10]. RNA quantity and purity were measured using NanoDrop 2000 (Thermo Fisher Scientific, Wilmington, DE, USA). RNA quality, yield and distribution were determined using an Agilent 2100 Bioanalyzer (Agilent Technologies, Santa Clara, CA, USA) [8, 11]. Healthy volunteers also provided 5 mL of blood samples, which were prepared using the above‐mentioned method.

### 2.3 Bioinformatic Analysis

The DEGs between the sepsis and control groups were identified by GEO2R, while setting the threshold of differential expression to the default standard (i.e., |log2 (fold change [FC])| > 1 and adjusted). Significance was defined as an adjusted *p*‐value < 0.05 to control for type I errors in multiple tests [12]. The Gene Ontology (GO) functional enrichment and Kyoto Encyclopedia of Genes and Genomes (KEGG) pathway enrichment analyses were performed to recognise the underlying biological functions of the DEGs identified in the previous step [13]. For GO functional annotation and KEGG pathway enrichment analysis, we used the WEB‐based Gene SeT AnaLysis Toolkit (WebGestalt), the web‐based Database for Annotation, Visualisation, and Integrated Discovery (DAVID) tool version 6.8 [12], and Metascape [14].

The GO functional annotation and KEGG pathway enrichment analysis revealed that the DEGs between the sepsis and control groups were mainly involved in the immune‐system‐related gene category [8]. Therefore, this study initially selected DEGs grouped into the immune response (immune system‐related) category and further narrowed down to those grouped into the necroptosis‐related category. The data have been deposited in NCBI's Gene Expression Omnibus and are available through the GEO Series accession number GES232753. Our previous studies described a more detailed methodology of bioinformatic analysis [1, 8].

### 2.4 Quantitative Real‐Time Polymerase Chain Reaction and Enzyme‐Linked Immunosorbent Assay—First Experimental Verification

As the first experimental verification, we performed quantitative real‐time polymerase chain reaction (qPCR) analyses in duplicate to evaluate the expression levels of necroptosis‐related DEGs. The reaction conditions were as follows: an initial step of 50°C for 2 min, denaturing at 95°C for 5 min, followed by 40 cycles of 95°C for 30 s and 58.5°C for 1 min. qPCR was performed on an ABI ViiA 7 Real‐Time PCR System (Applied Biosystems) and was followed by a melting curve analysis. Glyceraldehyde‐3‐phosphate dehydrogenase was selected as an internal control. The 2 − ∆∆CT algorithm (∆CT = Ct. target − Ct. reference) was employed for downstream data analysis [15].

To validate the results of the qPCR analysis, enzyme‐linked immunosorbent assay (ELISA) was performed to measure the levels of corresponding proteins related to the identified necroptosis‐related DEGs in the plasma of patients with sepsis and healthy volunteers. The glycogen phosphorylase L (PYGL) levels were measured using the Human PYGL ELISA Kit (Novus Biologicals, Centennial CO, USA). The NOD‐like receptor family pyrin domain containing 6 (NLRP6) and Cylindromatosis Lysine 63 deubiquitinase (CYLD) levels were measured using an ELISA kit (MyBiosource, San Diego, CA, USA). The Fas associated via death domain (FADD) levels were measured using the Human Protein FADD ELISA kit (CUSABIO, Wuhan, China). Tumour necrosis factor (TNF)‐α levels, Toll‐like receptor 3 (TLR3) and p53 were measured using a human ELISA kit (abcam, Cambridge, UK). The FasL levels were measured using the Human FasL ELISA Kit (R&D Systems, Minneapolis, USA). The experiments were conducted according to the manufacturer's instructions.

### 2.5 Cell Culture, Transfection and Western Blotting—Second Experimental Verification

As the second experimental validation, HUVECs (ATCC PCS‐100013), Jurkat cells (ATCC‐ TIB‐152) and THP‐1 cells (ATCC‐TIB‐202) were purchased from ATCC and cultured in the RPMI‐1640 medium (Thermo Fisher Scientific Inc.) containing 10% FBS (Hyclone; GE Healthcare Life Sciences) and 1% penicillin‐streptomycin solution in a cell incubator at 37°C and 5% CO_2_. For the experiment, HUVEC, Jurkat T and THP‐1 cells were transfected with the plasma from patients with sepsis and healthy volunteers for 24 h, which was followed by Western blotting.

Western blotting was used to measure the cellular protein levels. Total protein was extracted from the treated cells using a radio‐immunoprecipitation (RIPA) assay lysis buffer (Shanghai Yeasen Biotech Co. Ltd., Shanghai, China). A BCA protein detection kit (Beyotime Biotechnology Co., China) was employed to measure the concentration of supernatant protein. SDS‐PAGE was used to separate the whole protein and afterwards transport it to a polyvinylidene fluoride (PVDF) film. The membrane was sealed with 5% bovine serum albumin solution at room temperature for 1 h, and the closed membrane was incubated with primary antibodies at 4°C overnight and then incubated with goat anti‐rabbit secondary antibody for 1 h. The target protein signals were detected by using the enhanced chemiluminescence reagent and Optimax X‐ray Film Processor (Protec, Germany). Relative densitometric values of Western blots were calculated using Image Lab Ver. 6.0.1 software (Bio‐Rad Laboratories, Hercules, CA, USA).

### 2.6 Cytokine Array—Third Experimental Verification

**FIGURE 1:**
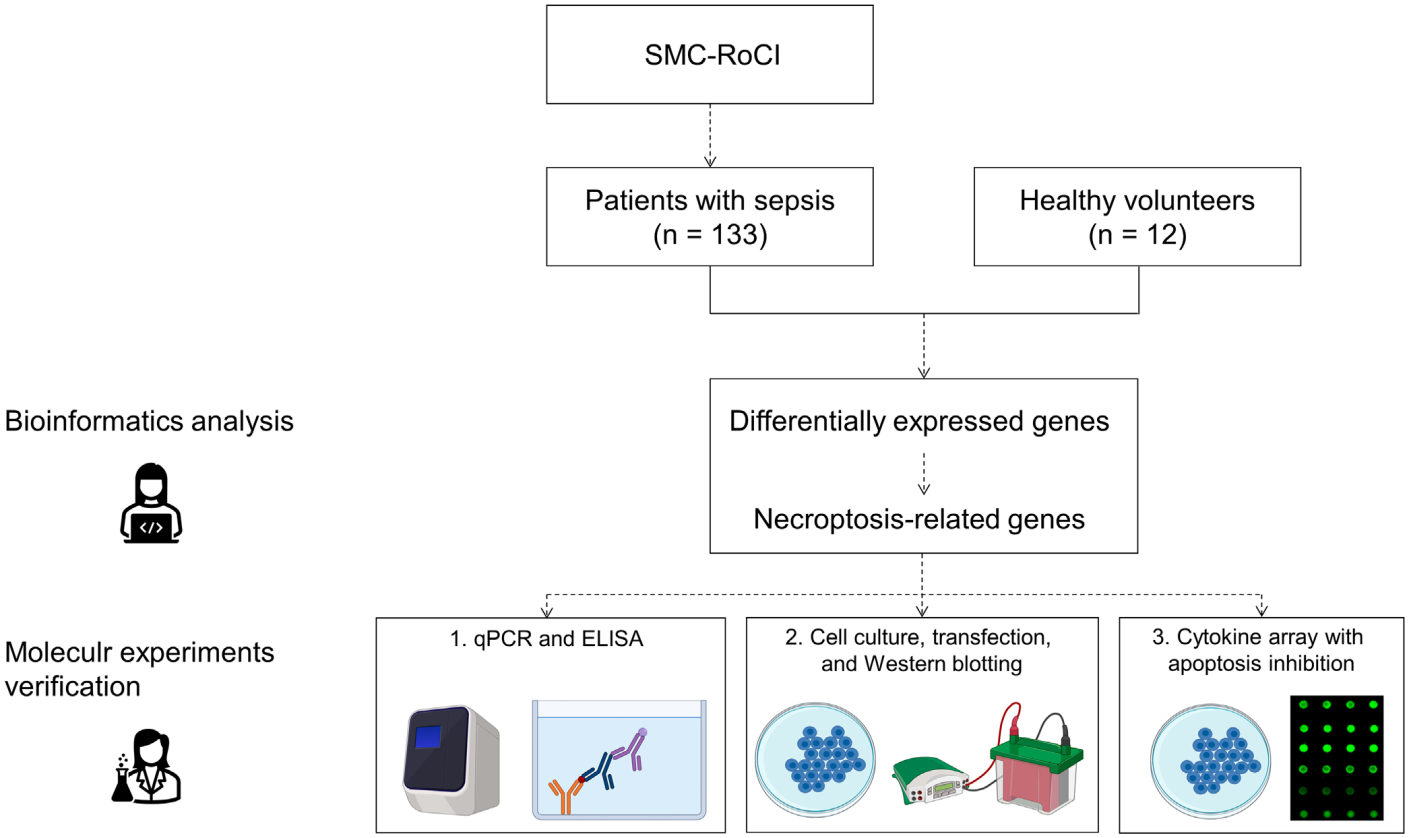
Study flow diagram. This figure was created with BioRender.com. ELISA, enzyme‐linked immunosorbent assay; qPCR, quantitative real‐time polymerase chain reaction; SMC‐RoCI, Samsung Medical Center Registry of Critical Illness.

Cytokine presence in culture media from transfected cells undergoing necroptosis was assessed using a Human Cytokine Array C1000 (RayBiotech, Norcross, GA, USA), followed by densitometric analysis (ImageJ, NIH, USA). For this experiment, the broad‐spectrum caspase inhibitor z‐VAD‐fmk was added to HUVEC, Jurkat T and THP‐1 cells that had been transfected with plasma from patients with sepsis. The z‐VAD‐fmk prevents apoptosis in many different cell types by inhibiting the caspase‐8 activity and consequently makes the transfected cells undergo necroptosis. The study flow is summarised in Figure 1.

### 2.7 Statistical Analysis

Data are presented as medians and interquartile ranges (IQRs) for continuous variables and as frequencies (percentages) for categorical variables. Continuous variables were compared using the Mann–Whitney *U* test because of non‐normality. A receiver‐operating characteristic (ROC) curve analysis was also used to evaluate whether the expression levels of proteins expressed by necroptosis‐related DEGs could differentiate between the sepsis and control groups and between the surviving and deceased patients in the sepsis group. We plotted ROC curves and calculated the area under the curve (AUC) of each DEG‐expressed protein. AUC values above 0.90 and 0.80 ≤ AUC values < 0.90 were interpreted as excellent and considerable diagnostic performances, respectively, in this study [16]. All tests were two‐sided, and the *p*‐value < 0.05 was considered significant. Data were analysed using STATA version 16 (Stata Corp., College Station, TX, USA).

## 3 Results

### 3.1 Clinical Characteristics of Patients With Sepsis

During the study period, 133 patients with sepsis were enrolled with a median age of 66 (58–73) years; 90 (67.7%) patients were male. Figure S1 depicts a study population selection flow chart. Any malignancies, including solid organ and hematologic, were the most common comorbidity (38.3%), followed by diabetes mellitus (30.8%) and chronic obstructive pulmonary disease (12.0%). When the patients were admitted to the intensive care unit, 60 (45.1%) and 69 (51.9%) patients required mechanical ventilation and vasopressor support, respectively. Regarding the severity of illness upon admission, the median Simplified Acute Physiology Score 3 was 55 (47–63), the Acute Physiology and Chronic Health Evaluation II score was 24 (20–30), and the Sequential Organ Failure Assessment score was 9 (7–11). Regarding in‐hospital mortality, 33 (24.8%) died and 100 (75.2%) survived (Table 1).

**TABLE 1:** Clinical characteristics of the patients with sepsis.

	Sepsis patients (*n* = 133)
Age, years	66 (58–73)
Male	90 (67.7)
Comorbidities	
All malignancies	51 (38.3)
Solid organ malignancies	35 (26.3)
Hematologic malignancies	16 (12.0)
Diabetes mellitus	41 (30.8)
Chronic obstructive pulmonary disease	16 (12.0)
Chronic kidney disease	10 (7.5)
Myocardial infarction	8 (6.0)
Congestive heart failure	7 (5.3)
Cerebrovascular disease	8 (6.0)
Chronic liver disease	11 (8.3)
Charlson Comorbidity Index	2 (1–3)
Clinical status on ICU admission	
Mechanical ventilation	60 (45.1)
Vasopressor support	69 (51.9)
Laboratory findings	
PaO_2_/FiO_2_ ratio	216 (135–323)
CRP, mg/dL	12.5 (5.8–24.5)
Lactate, mg/dL	3.0 (2.0–4.4)
Severity of illness on ICU admission	
SAPS 3	55 (47–63)
APACHE II score	24 (20–30)
SOFA score	9 (7–11)
Outcome	
In‐hospital mortality	33 (24.8)

*Note:* Values are presented as medians (interquartile ranges) or numbers (percentages).

Abbreviations: APACHE II, Acute Physiology and Chronic Health Evaluation II; CRP, C‐reactive protein; ICU, intensive care unit; PaO_2_/FiO_2_ ratio, ratio of arterial oxygen pressure to fractional inspired oxygen; SAPS 3, Simplified Acute Physiology Score 3; SOFA, Sequential Organ Failure Assessment.

### 3.2 Necroptosis‐Related DEGs Identified by Bioinformatic Analysis

Initially, 422 DEGs were identified between 133 patients with sepsis and 12 healthy volunteers. Among the identified 422 genes, we subsequently selected 93 genes that were grouped into the immune response category. The gene symbols and FCs of all 93 immune response‐related and 32 necroptosis‐related DEGs are also provided in Table S1. Furthermore, the 93 genes were narrowed down to eight genes that were also related to the necroptosis pathway, including five upregulated (PYGL, TNF, CYLD, FADD and TLR3) and three downregulated (TP53, FASLG and NLRP6) genes (Table 2).

**TABLE 2:** Comparison of eight differentially expressed genes, grouped into both immune system‐related and necroptosis‐related categories, plus corresponding proteins between the sepsis and control groups.

	Entrez ID	Gene symbol	Fold change	Corresponding protein
Five upregulated genes	17,408	PYGL	10.39	PYGL
	21,992	TNF	7.90	TNF‐α
	4455	CYLD	7.86	CYLD
	5962	FADD	6.51	FADD
	7098	TLR3	4.48	TLR3
Three downregulated genes	22,119	TP53	0.19	p53
	6369	FASLG	0.26	FasL
	14,583	NLRP6	0.33	NLRP6

Abbreviations: CYLD, Cylindromatosis Lysine 63 deubiquitinase; FADD, Fas associated via death domain; FASLG, Fas ligand; NLRP6, NLR family pyrin domain containing 6; PYGL, glycogen phosphorylase L; TLR3, toll‐like receptor 3; TNF, tumour necrosis factor; TP53, tumour protein p53.

### 3.3 Quantitative Real‐Time PCR and ELISA—First Experimental Verification

Necroptosis‐related DEG expression levels were compared between the sepsis (*n* = 133) and control (*n* = 12) groups using qPCR. Among the eight DEGs, the expression levels of five upregulated genes, including PYGL, TNF, CYLD, FADD and TLR3, were significantly higher in the sepsis group than in the control group (*p* < 0.001 for all); additionally, three downregulated genes, including TP53, FASLG and NLRP6, showed significantly lower expression levels in the sepsis group than in the healthy volunteers (*p* < 0.001 for all) (Table 3). A comparison of the necroptosis‐related DEG expression levels between the deceased (*n* = 33) and surviving (*n* = 100) among those with sepsis was also conducted using qPCR. Three upregulated genes, including PYGL, TNF and CYLD, showed significantly higher expression levels in the deceased patients than in the surviving patients (*p* < 0.001 for all), whereas one downregulated gene, TP53, showed significantly lower expression levels in the deceased patients than in the surviving patients (*p* < 0.001) (Table 3).

**TABLE 3:** Comparison of the expression levels of necroptosis‐related differentially expressed genes between the sepsis and control groups and between the deceased and surviving patients in the sepsis group (quantitative real‐time polymerase chain reaction analysis).

	Gene symbol	Sepsis patients (*n* = 133)	Healthy volunteers (*n* = 12)	*p*
Patients with sepsis vs. healthy volunteers				
Upregulated	PYGL	17.9 (14.4–20.4)	7.5 (7.3–7.7)	< 0.001
	TNF	6.7 (4.2–8.3)	2.9 (2.3–3.5)	< 0.001
	CYLD	7.3 (4.9–9.4)	2.3 (2.1–2.4)	< 0.001
	FADD	6.4 (6.2–6.7)	2.6 (2.4–3.0)	< 0.001
	TLR3	5.6 (5.5–5.8)	2.0 (2.0–2.1)	< 0.001
Downregulated	TP53	0 (0–0.1)	0.2 (0.2–0.2)	< 0.001
	FASLG	0 (0–0)	0.1 (0.1–0.1)	< 0.001
	NLRP6	0 (0–0)	0.1 (0.1–0.1)	< 0.001

	Gene symbol	Dead patients (*n* = 33)	Survived patients (*n* = 100)	*p*
The dead vs. survived among patients with sepsis				
Upregulated	PYGL	21.1 (20.1–22.2)	16.9 (13.8–18.7)	< 0.001
	TNF	8.9 (8.2–9.5)	5.9 (3.9–7.4)	< 0.001
	CYLD	11.1 (7.9–12.2)	6.2 (4.4–8.4)	< 0.001
	FADD	6.3 (6.2–6.7)	6.6 (6.2–6.7)	0.487
	TLR3	5.6 (5.6–5.8)	5.6 (5.5–5.8)	0.890
Downregulated	TP53	0.1 (0.1–0.1)	0 (0–0)	< 0.001
	FASLG	0 (0–0)	0 (0–0)	0.981
	NLRP6	0 (0–0)	0 (0–0)	0.823

*Note:* Values are presented as medians (interquartile ranges), and the two groups were compared using the Mann–Whitney *U* test.

Abbreviations: CYLD, Cylindromatosis Lysine 63 deubiquitinase; FADD, Fas associated via death domain; FASLG, Fas ligand; NLRP6, NLR family pyrin domain containing 6; PYGL, glycogen phosphorylase L; TLR3, toll‐like receptor 3; TNF, tumour necrosis factor; TP53, tumour protein p53.

Then, we compared the levels of the corresponding proteins to the necroptosis‐related DEGs between the sepsis and control groups using ELISA. The levels of the corresponding proteins to the five upregulated DEGs (PYGL, TNF‐α, CYLD, FADD and TLR3) were significantly higher in the sepsis group than in the control group (*p* < 0.001 for all); additionally, the levels of corresponding proteins to the three downregulated DEGs (p53, FasL and NLRP6) were significantly lower in the sepsis group than in the control group (*p* < 0.001 for all) (Table 4). We also compared the levels of corresponding proteins to necroptosis‐related DEGs between the deceased and surviving patients among patients with sepsis using ELISA. The levels of corresponding proteins to the five upregulated DEGs (PYGL, TNF‐α, CYLD, FADD and TLR3) were significantly higher in the deceased patients than in the surviving patients (*p* < 0.001 for all); furthermore, the levels of corresponding proteins to the three downregulated DEGs (p53, FasL and NLRP6) were also significantly lower in the deceased patients than in the surviving patients (*p* < 0.001 for all) (Table 4).

**TABLE 4:** Comparison of the levels of proteins related to necroptosis‐related differentially expressed genes between the sepsis and control groups and between the deceased and surviving patients in the sepsis group (ELISA analysis).

	Protein	Sepsis patients (*n* = 133)	Healthy volunteers (*n* = 12)	*p*
Patients with sepsis vs. healthy volunteers				
Upregulated	PYGL, ng/mL	4.8 (2.2–7.4)	1.3 (1.2–1.4)	< 0.001
	TNF‐α, pg/mL	330.0 (199.0–408.0)	89.5 (80.3–114.8)	< 0.001
	CYLD, ng/mL	6.0 (4.4–8.0)	2.3 (2.1–2.4)	< 0.001
	FADD, pg/mL	563.0 (329.0–865.0)	268.8 (227.9–309.5)	< 0.001
	TLR3, pg/mL	6358.0 (4884.0–7966.0)	3071.3 (2518.6–3459.3)	< 0.001
Downregulated	p53, pg/mL	55.0 (25.0–88.0)	415.0 (400.0–419.5)	< 0.001
	FasL, ng/mL	600.0 (290.0–870.0)	1841.5 (1785.5–1920.0)	< 0.001
	NLRP6, pg/mL	3.0 (1.4–3.9)	8.8 (8.8–9.1)	< 0.001

	Protein	Dead patients (*n* = 33)	Survived patients (*n* = 100)	*p*
The dead vs. survived among patients with sepsis				
Upregulated	PYGL, ng/mL	9.0 (7.4–10.0)	3.6 (1.7–5.8)	< 0.001
	TNF‐α, pg/mL	464.0 (403.0–504.0)	262.0 (176.5–362.5)	< 0.001
	CYLD, ng/mL	8.4 (7.4–9.4)	5.1 (4.1–6.9)	< 0.001
	FADD, pg/mL	1243.0 (726.0–1793.0)	419.5 (288.5–652.5)	< 0.001
	TLR3, pg/mL	8207.0 (7756.0–9064.0)	5568.0 (4622.0–7171.0)	< 0.001
Downregulated	p53, pg/mL	13.3 (7.0–23.0)	72.5 (50.0–100.00)	< 0.001
	FasL, ng/mL	123.0 (83.0–280.0)	705.0 (495.0–998.5)	< 0.001
	NLRP6, pg/mL	1.3 (0.9–1.5)	3.4 (2.7–4.1)	< 0.001

*Note:* Values are presented as medians (interquartile ranges), and the two groups were compared using the Mann–Whitney *U* test.

Abbreviations: CYLD, Cylindromatosis Lysine 63 deubiquitinase; ELISA, enzyme‐linked immunosorbent assay; FADD, Fas associated via death domain; FasL, Fas ligand; NLRP6, NLR family pyrin domain containing 6; PYGL, glycogen phosphorylase L; TLR3, toll‐like receptor 3; TNF, tumour necrosis factor.

**FIGURE 2:**
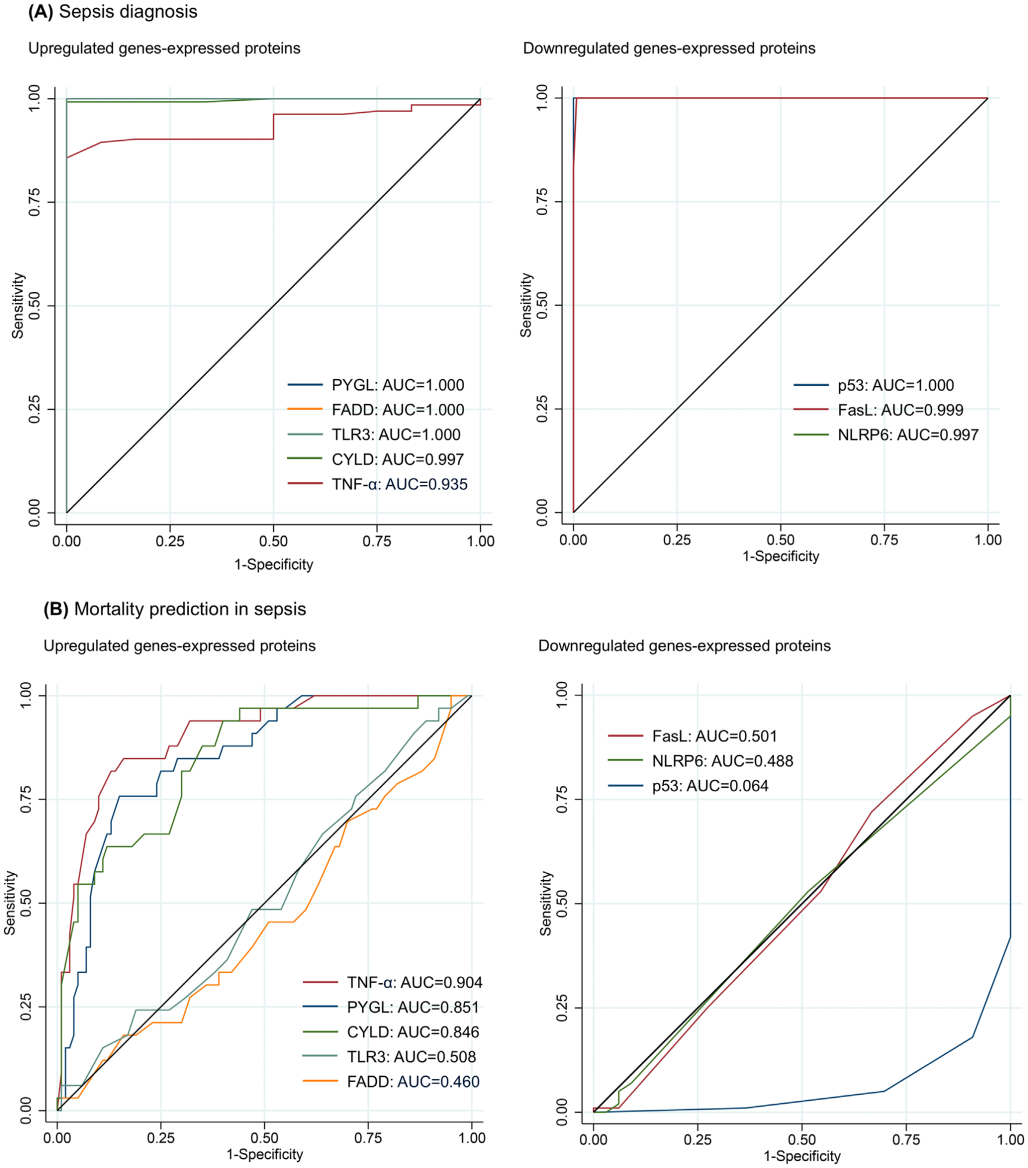
Graphs showing receiver operating characteristic curves of proteins corresponding to the upregulated and downregulated necroptosis‐related genes for diagnosing sepsis in all study participants (*n* = 145) (A) and predicting mortality in patients with sepsis (*n* = 133) (B). Protein levels were measured using an enzyme‐linked immunosorbent assay. AUC, area under the curve; CYLD, Cylindromatosis Lysine 63 deubiquitinase; FADD, Fas associated via death domain; FasL, Fas ligand; NLRP6, NLR family pyrin domain containing 6; PYGL, glycogen phosphorylase L; TLR3, toll‐like receptor 3; TNF, tumour necrosis factor.

Notably, the levels of corresponding proteins to the necroptosis‐related DEGs, comprising five upregulated and three downregulated genes, showed excellent accuracy in diagnosing sepsis (AUC, 0.935–1.000). Regarding the prediction of mortality in patients with sepsis, the levels of corresponding proteins to the three upregulated necroptosis‐related genes showed excellent or considerable accuracy (AUC; TNF‐α = 0.904, PYGL = 0.851 and CYLD = 0.846) (Figure 2).

### 3.4 Cell Culture, Transfection and Western Blotting—Second Experimental Verification

**FIGURE 3:**
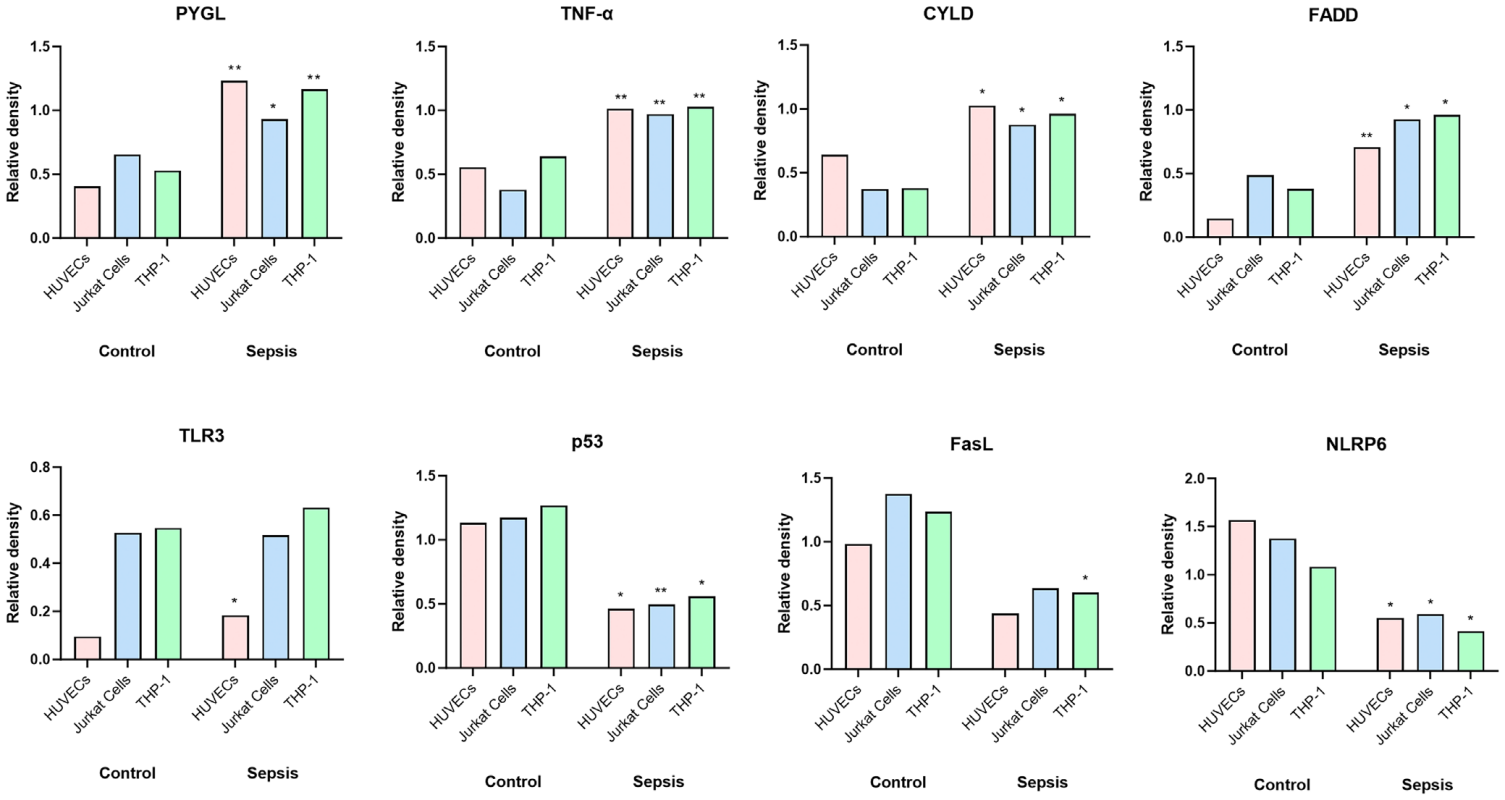
Quantification of corresponding proteins to the eight necroptosis‐related differentially expressed genes between patients with sepsis and healthy volunteers. The protein expression levels were measured in three cells, comprising HUVEC, Jurkat T and THP‐1 cells, with media transfected with plasma from sepsis patients and healthy volunteers for 24 h. Statistical significance was determined by comparing the protein expression level in each cell medium between patients with sepsis and healthy volunteers (*p < 0.05 and **p < 0.01). CYLD, Cylindromatosis Lysine 63 deubiquitinase; FADD, Fas associated via death domain; FasL, Fas ligand; NLRP6, NLR family pyrin domain containing 6; PYGL, glycogen phosphorylase L; TLR3, toll‐like receptor 3; TNF, tumour necrosis factor.

In three cells, comprising HUVECs, Jurkat T and THP‐1 cells, the culture media were transfected with plasma from patients with sepsis and healthy volunteers for 24 h, and the levels of corresponding proteins to the eight necroptosis‐related DEGs were compared between the sepsis and control groups (the final product of western blotting is provided in Figure S2). The protein expression levels of four upregulated necroptosis‐related DEGs (PYGL, TNF‐α, CYLD and FADD) were significantly higher in the sepsis group than in the control group in all three cells, whereas the TLR3 level was only significantly higher in HUVEC cells (Figure 3). Regarding the downregulated necroptosis‐related DEGs, the p53 and NLRP6 levels were substantially lower in the sepsis group than in the control group in all three cells; however, the FasL level was only significantly lower in the THP‐1 cell (Figure 3).

### 3.5 Cytokine Array—Third Experimental Verification

**FIGURE 4:**
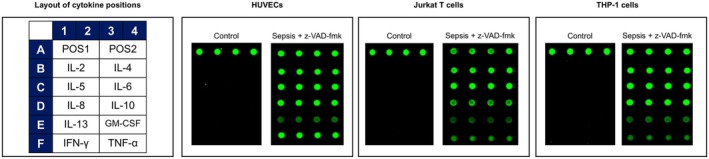
Cytokine array to assess cytokine presence in three culture media from transfected cells undergoing necroptosis by inhibiting the caspase‐8 activity and consequently preventing apoptosis. GM‐CSF, granulocyte‐macrophage colony‐stimulating factor; IFN, interferon; IL, interleukin; POS, positive control spot; TNF, tumour necrosis factor.

Caspase‐8 is the initiator caspase of extrinsic apoptosis and inhibits necroptosis, which is mediated by RIPK3 and MLKL [17]. Therefore, while inhibiting the caspase‐8 activity and consequently preventing apoptosis, we performed cytokine arrays in three cell culture media, including HUVEC, Jurakat T and THP‐1 cells, transfected with plasma from patients with sepsis. The presence of interleukin (IL)‐2, IL‐4, IL‐5, IL‐6, IL‐8, IL‐10, TNF‐α and interferon‐γ was significantly more evident in all three cell culture media transfected with plasma from sepsis patients than in controls, indicating that the transfected cells potentially underwent necroptosis (Figure 4).

## 4 Discussion

Eight necroptosis‐related DEGs, comprising five upregulated (PYGL, TNF, CYLD, FADD and TLR3) and three downregulated (TP53, FASLG and NLRP6) DEGs, were identified by bioinformatic analysis between patients with sepsis and healthy volunteers. The results of the bioinformatic analysis were verified by three‐step molecular experiments: (1) qPCR and ELISA; (2) cell culture, transfection and Western blotting; and (3) cytokine array with apoptosis inhibition. In addition to the sepsis diagnosis, the necroptosis‐related DEGs played major roles in predicting mortality among patients with sepsis. Therefore, the present study indicates that necroptosis is potentially a key mechanism of sepsis.

Notably, our study results confirming necroptosis as a crucial mechanism of sepsis are meaningful because several studies have recently revealed that organ dysfunction in sepsis is not caused by the pathogen itself, but by a dysregulated host response to infection [9, 18, 19]. Consistent with our findings, the RIPK3 deficiency conferred protection against lethal systemic inflammation and reduced circulating damage‐associated molecular patterns (DAMPs) in a mouse model of TNF‐induced systemic inflammatory response [2, 20]. Additionally, clinical studies have shown associations between the levels of necroptosis mediators, including receptor‐interacting kinase 1, RIPK3 and MLKL, and the severity and outcomes of sepsis [21, 22, 23, 24]. Beyond the previous basic and clinical studies, this study has value as it establishes the mechanistic link between necroptosis and sepsis by initially performing bioinformatic analysis and then molecular experiments.

**FIGURE 5:**
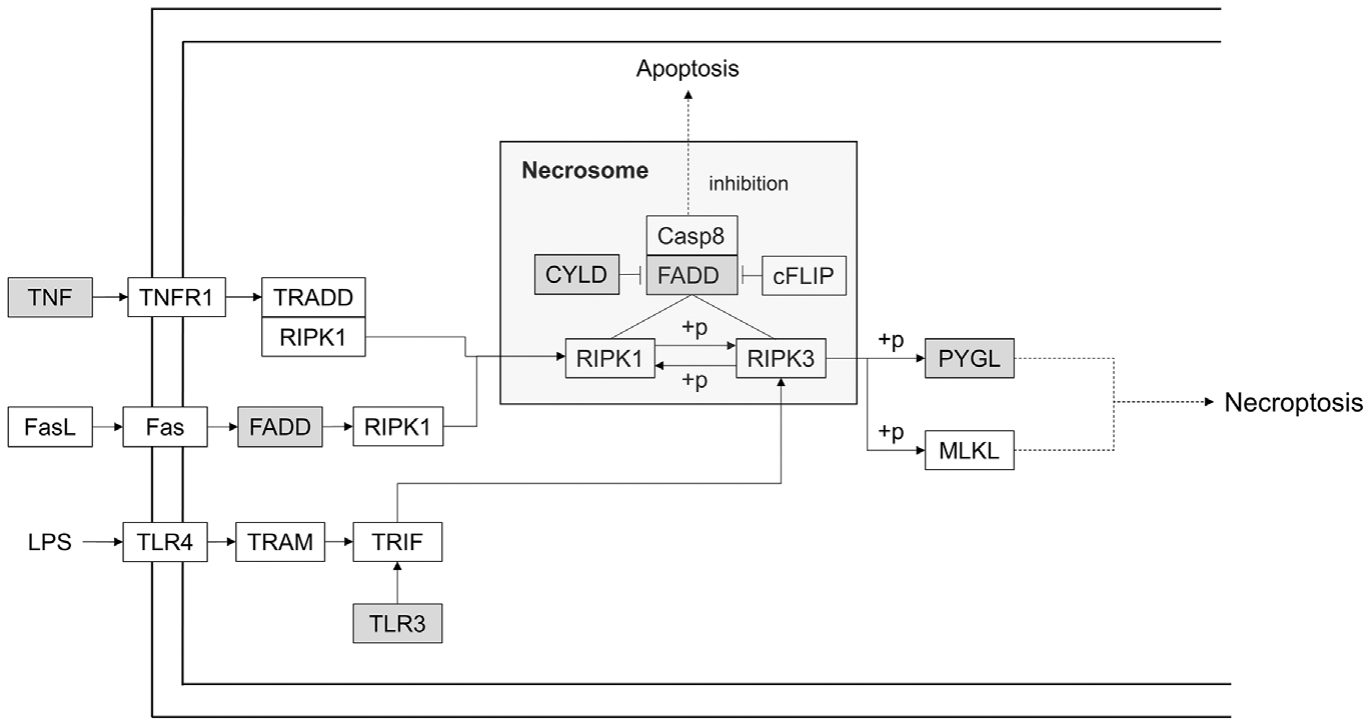
Schematic representation of the necroptosis pathway. Five upregulated necroptosis‐related differentially expressed genes that were identified and verified in this study are presented in grey colour. TNFR1 activation by TNF results in the complex formation between RIPK1 and TRADD. Under conditions of caspase‐8 inhibition, necroptosis occurs through the formation of the necrosome, which is a three‐protein complex (RIPK1, RIPK3 and FADD). The necrosome activates MLKL and phospho‐MLKL oligomerizes and translocates into the plasma membrane, where it causes membrane rupture and release of damage‐associated molecular patterns. Alternatively, the activation of Fas or receptor/ligand interaction, such as TLR3/LPS, also results in necroptosis. Casp8, caspase‐8; cFLIP, cellular FLICE (FADD‐like IL‐1β‐converting enzyme)‐inhibitory protein; FADD, Fas‐associated death domain; FasL, Fas ligand; LPS, lipopolysaccharide; MLKL, mixed‐lineage kinase domain‐like; PYGL, glycogen phosphorylase L; RIPK1, receptor‐interacting protein kinase 1; RIPK3, receptor‐interacting protein kinase 3; TLR3, toll‐like receptor 3; TLR4, toll‐like receptor 4; TNF, tumour necrosis factor; TNFR1, TNF receptor 1; TRADD, TNFR1‐associated death domain; TRAM, TRIF‐related adaptor molecule; TRIF, TIR domain‐containing adaptor protein.

Figure 5 depicts a schematic representation of the necroptosis pathway. Briefly, TNF receptor 1 activation by TNF results in the complex formation between RIPK1 and TNF receptor 1‐associated death domain. Under the condition of caspase‐8 activity inhibition, necroptosis occurs through the formation of the necrosome, a three‐protein complex (RIPK1, RIPK3 and FADD). The necrosome activates MLKL, and phospho‐MLKL oligomerizes and translocates into the plasma membrane, where it causes membrane rupture and release of DAMPs [2, 25, 26, 27]. Alternately, the activation of Fas or receptor/ligand interaction, including TLR3/lipopolysaccharide, also results in necroptosis [2]. Remarkably, the present study identified and verified five DEGs related to the execution molecules of necroptosis. Indeed, we did not include all necroptosis‐related genes in our analysis. For example, although RIPK3 is a key molecule of necroptosis, this gene was not included in our study because we only selected necroptosis‐related DEGs among the initially chosen immune system‐related DEGs, which are most highly enriched in patients with sepsis, as the expansion of our previous study [8].

As a part of the cell culture experiment, we demonstrated that cytokines were evident in the culture media from the transfected cells undergoing necroptosis by inhibiting the activity of caspase‐8 and consequently preventing apoptosis. Additionally, the downregulated DEGs included TP53 that is related to the p53 pathway, which is a very well‐known part of apoptotic cell death [28, 29]. These results imply that necroptosis is more likely to influence organ damage in sepsis than apoptosis. In agreement with a previous concept [25], TNF was upregulated, but the FasL was downregulated in the present study; therefore, the TNF pathway seemed the main trigger for enhancing the proinflammatory response and consequent necroptosis in sepsis. Furthermore, in the plasma mitochondrial DNA, a potent DAMP, the copy number was highly correlated with the essential necroptosis mediators, including RIPK3, MLKL and HMGB1, suggesting that mitochondrial DNA propagates necroptosis and increases the mortality rate due to sepsis in our previous study [1]. Taken together, this phenomenon can be explained through the concept of an ‘auto‐amplification loop’—following an initial event of regulated cell death (necroptosis), regulated cell death and inflammation can induce each other and drive a local auto‐amplification loop that leads to exaggerated cell death and organ failure [30].

Notably, our study identified necroptosis‐related DEGs and proteins that can serve as biomarkers to diagnose sepsis and predict its mortality and therapeutic targets. The levels of five proteins expressed by upregulated necroptosis‐related DEGs demonstrated excellent accuracy in diagnosing sepsis and predicting the mortality of patients with sepsis, according to the results of the AUC analyses. These results clearly showed the role of necroptosis‐related DEGs identified by this study in diagnosing and determining the prognosis of sepsis. Moreover, regarding a potential therapeutic target, in this study, the levels of the CYLD gene and related protein expression were well‐correlated with sepsis diagnosis and mortality, which was also demonstrated in previous studies [31, 32, 33]; therefore, CYLD could be a future therapeutic target by controlling cell death in patients with sepsis. In the same vein, a recently published study adopting machine learning also revealed necroptosis hub genes by comparing the data between patients with sepsis and healthy volunteers, which suggested great potential of the genes in predicting prognosis and personalised immunotherapy for sepsis [34].

The major strength of this study was that it identified necroptosis‐related DEGs between patients with sepsis and healthy volunteers and verified the findings by several molecular experiments comprehensively, thereby confirming the crucial role of necroptosis in sepsis. Nonetheless, this study has some limitations. First, a relatively small number of the study population may warrant future studies that include a larger sample size to confirm our findings. Second, as this study was conducted in Korea, more studies involving other ethnic groups need to be performed to generalise our study results. Third, the subset of patients with sepsis had other comorbidities, such as malignancies, which might have influenced the DEGs between patients with sepsis and healthy volunteers. Fourth, as this study computed the ROC curve analyses without a validation dataset, there is a possibility of overfitting. Fifth, age‐ and sex‐matching were not done between patients with sepsis and healthy volunteers.

## 5 Conclusions

Eight necroptosis‐related DEGs were identified in patients with sepsis through bioinformatic analysis and verified by molecular experiments, including cytokine arrays with apoptosis inhibition, which suggests that necroptosis may be a key mechanism of sepsis. Future studies are warranted to confirm our findings.

## Author Contributions

**Hayoung Choi:** data curation (lead), formal analysis (lead), investigation (lead), methodology (equal), resources (equal), writing – original draft (lead), writing – review and editing (equal). **Jin Young Lee:** data curation (lead), formal analysis (lead), investigation (lead), methodology (equal), resources (equal), visualization (equal), writing – original draft (lead), writing – review and editing (equal). **Hongseok Yoo:** formal analysis (equal), investigation (equal), methodology (equal), writing – review and editing (equal). **Kyeongman Jeon:** conceptualization (lead), data curation (equal), formal analysis (equal), funding acquisition (lead), investigation (equal), methodology (equal), resources (equal), supervision (lead), validation (equal), writing – original draft (lead), writing – review and editing (lead).

## Ethics Statement

The study was performed in accordance with the guidelines stipulated in the Declaration of Helsinki. The Ethics Committee of Samsung Medical Center (IRB no. 2013–12‐033) reviewed and approved the study.

## Consent

Written informed consent was obtained from the patients or their legally authorised representatives prior to enrolment.

## Conflicts of Interest

The authors declare no conflicts of interest.

## Supporting information


Appendix S1.

Appendix S2.

Appendix S3.

## Data Availability

The data that support the study findings are available on request from the corresponding author. The data are not publicly available due to privacy or ethical restrictions.
