# Supplementary figures and images for: Identification and Verification of Necroptosis‐Related Genes in Patients With Sepsis by Bioinformatic Analysis and Molecular Experiments
Source: J Cell Mol Med. 2025 May 3;29(9):e70582. doi: 10.1111/jcmm.70582 (PMC12049152; doi:10.1111/jcmm.70582)

**Supplementary Figure S1.** Study population selection flow chart

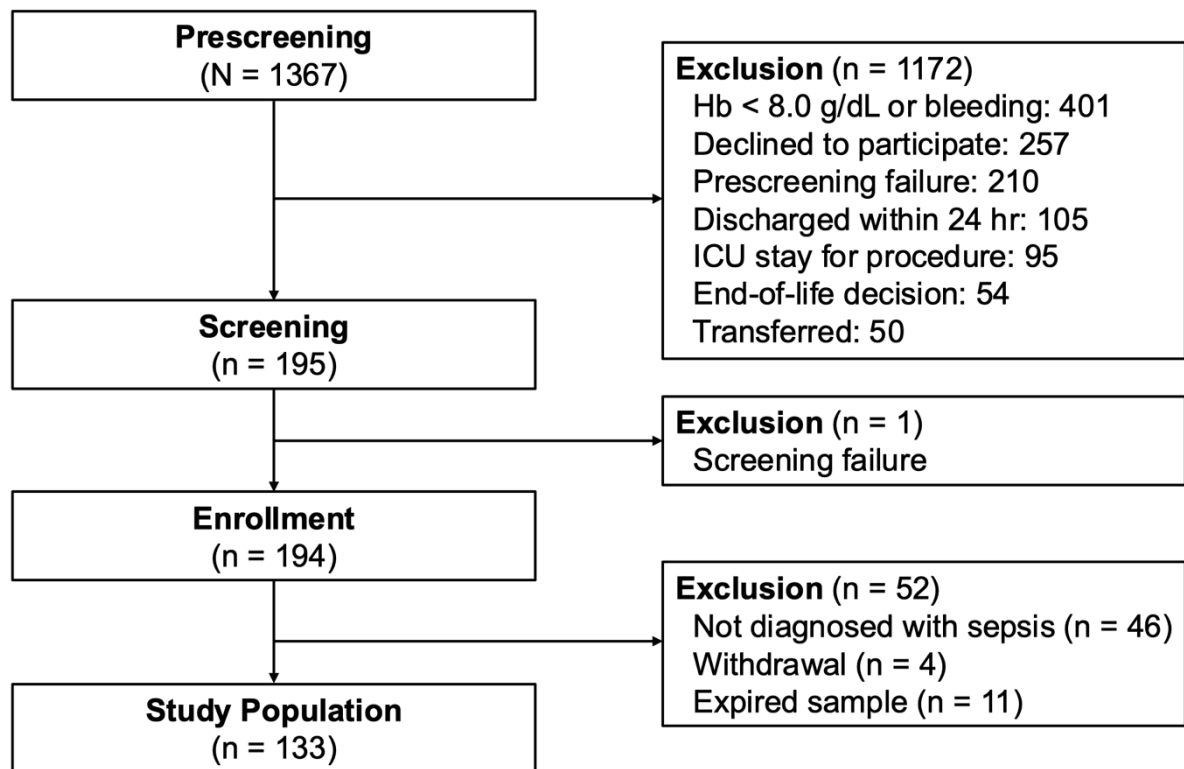

Supplement: Supplementary file 1 — Appendix S1. [file JCMM-29-e70582-s003.pdf]
