# Supplementary material for: Identification and Verification of Necroptosis‐Related Genes in Patients With Sepsis by Bioinformatic Analysis and Molecular Experiments
Source: J Cell Mol Med. 2025 May 3;29(9):e70582. doi: 10.1111/jcmm.70582 (PMC12049152; doi:10.1111/jcmm.70582)
Supplement: Supplementary file 2 — Appendix S2. [file JCMM-29-e70582-s002.pdf]

Supplementary Table S1. Comparison of differentially expressed genes, grouped into immune system-related and necroptosis-related categories, between the sepsis and control groups

1) Genes grouped into immune system-related category

Gene	Fold change	Gene	Fold change	Gene	Fold change
S100A8	16.168	MSRB1	2.293	CD81	0.345
VNN1	7.827	LILRB3	2.258	TP53	0.339
HMGB2	7.228	IL17RA	2.223	HLA-DRB1	0.325
S100A9	5.433	HIST1H2BC	2.196	CD8B	0.311
ANXA1	5.416	CD164	2.156	HLA-DPB1	0.310
BNIP3L	4.673	CASP1	2.148	CD6	0.308
APOBEC3A	4.607	NMI	2.144	SIT1	0.299
LILRA5	4.491	ACTR3	2.111	CTSW	0.299
CR1	4.459	AKIRIN2	2.090	PI3	0.298
CD55	4.287	IL10RB	2.070	CD74	0.296
SRPK1	4.055	MAP3K5	2.046	CD27	0.288
IFNGR1	3.968	RPL13A	0.491	HLA-DPA1	0.285
RIOK3	3.948	RPS19	0.484	LEF1	0.284
TLR8	3.609	SIGLEC10	0.468	LCK	0.271
PADI4	3.607	LFNG	0.466	CD247	0.252
CD58	3.530	CCL5	0.464	CD3E	0.248
LILRA6	3.336	IL27RA	0.458	PRF1	0.242
NLRC4	3.176	POU2F2	0.457	ZAP70	0.240
TNFSF10	3.011	HLA-DRA	0.450	SPON2	0.222
PTPRC	2.974	TRIM28	0.440	CD7	0.216
FPR2	2.967	LIME1	0.440	GZMM	0.215
SLA	2.890	CSF1R	0.438	CD79B	0.213
FCER1G	2.884	HLA-DMB	0.414	GNLY	0.206
DYSF	2.882	HLA-DMA	0.402	CD22	0.201
B2M	2.853	ST6GAL1	0.397	TCF7	0.199
RAB27A	2.644	SPN	0.385	VPREB3	0.176
TLR6	2.552	ETS1	0.378	NCR3	0.174
TRIM25	2.483	IL7R	0.375	CCR7	0.153
TLR1	2.469	IL32	0.354	PLD4	0.140
ITGAM	2.446	CD4	0.354	HLA-DQB2	0.129
FCGR3B	2.307	SKAP1	0.348	CD79A	0.119

2) Genes grouped into necroptosis-related category

Gene	Fold change
PYGL	10.394
MLKL	9.987
PELI1	8.766
RIPK3	8.441
IPMK	8.064
RIPK1	7.972
TNF	7.902
CYLD	7.865
YBX3	7.590
FADD	6.507
BIRC2	5.669
CFLAR	5.546
TLR3	4.481
OGT	4.295
CAV1	3.292
ARHGEF2	2.288
ZBP1	4.263
FZD9	0.992
TRPM7	0.742
SPATA2	0.733
RBCK1	0.728
CASP8	0.723
DNM1L	0.719
SLC25A4	0.653
ITPK1	0.548
PPIF	0.485
BIRC3	0.398
BOK	0.369
PGAM5	0.359
NLRP6	0.331
FASLG	0.258
TP53	0.193
