# Supplementary material for: Identification and Verification of Necroptosis‐Related Genes in Patients With Sepsis by Bioinformatic Analysis and Molecular Experiments
Source: J Cell Mol Med. 2025 May 3;29(9):e70582. doi: 10.1111/jcmm.70582 (PMC12049152; doi:10.1111/jcmm.70582)

Supplementary Figure S2. Final product of western blotting

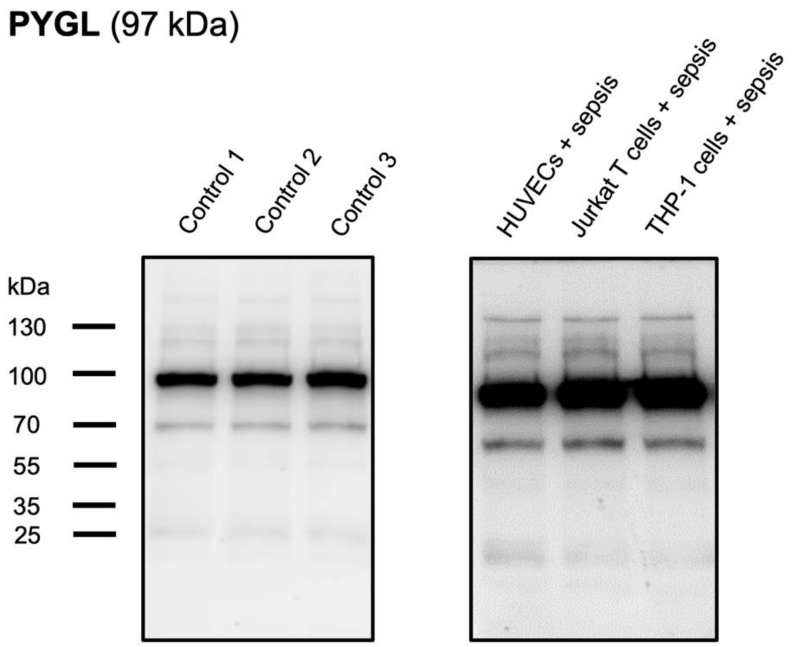

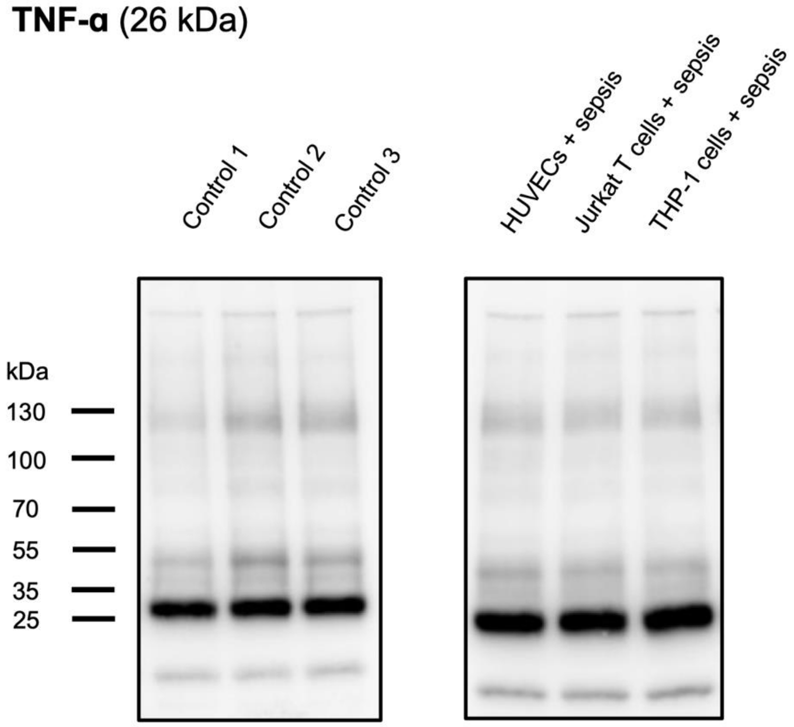

CYLD (107 kDa)

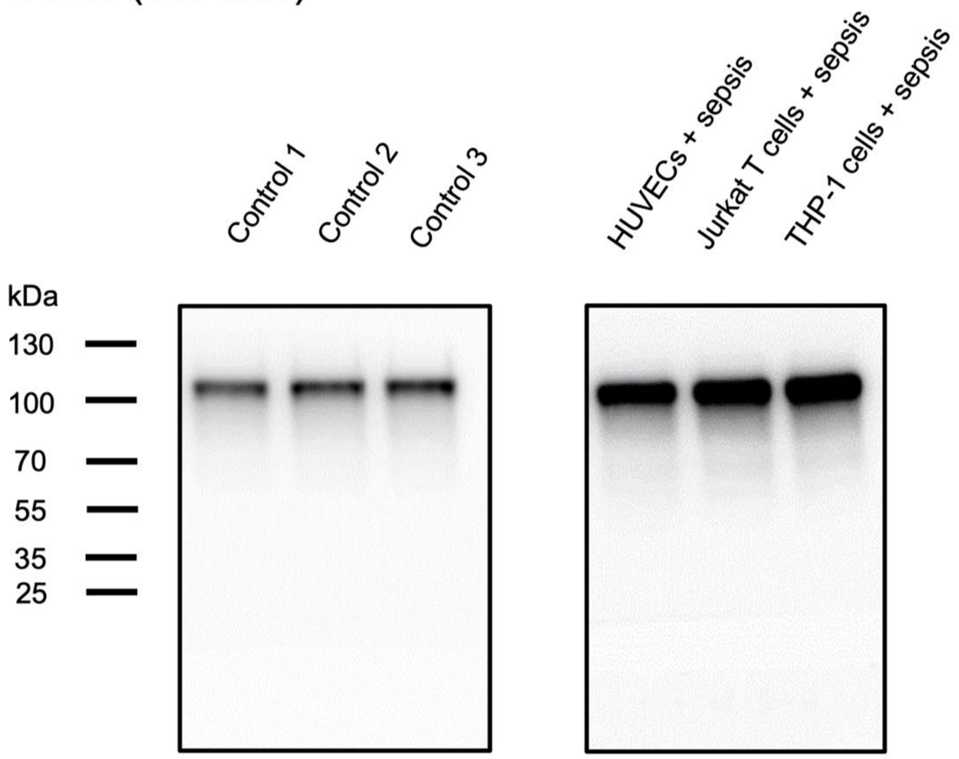

FADD (23 kDa)

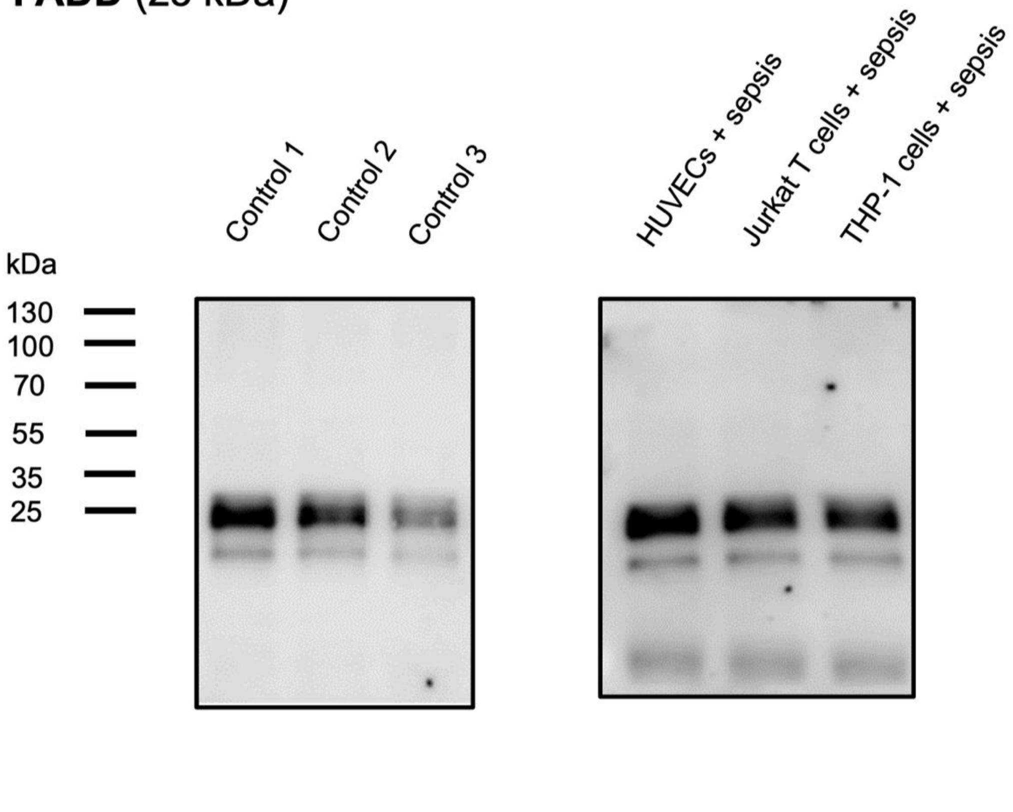

TLR3 (95 kDa)

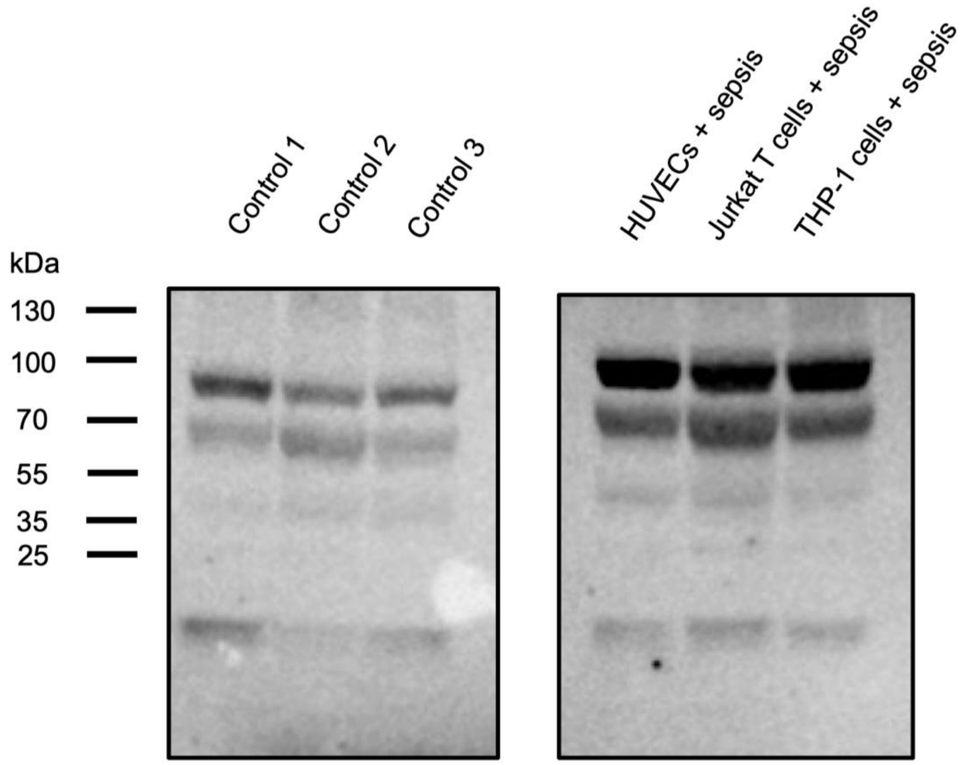

p53 (53 kDa)

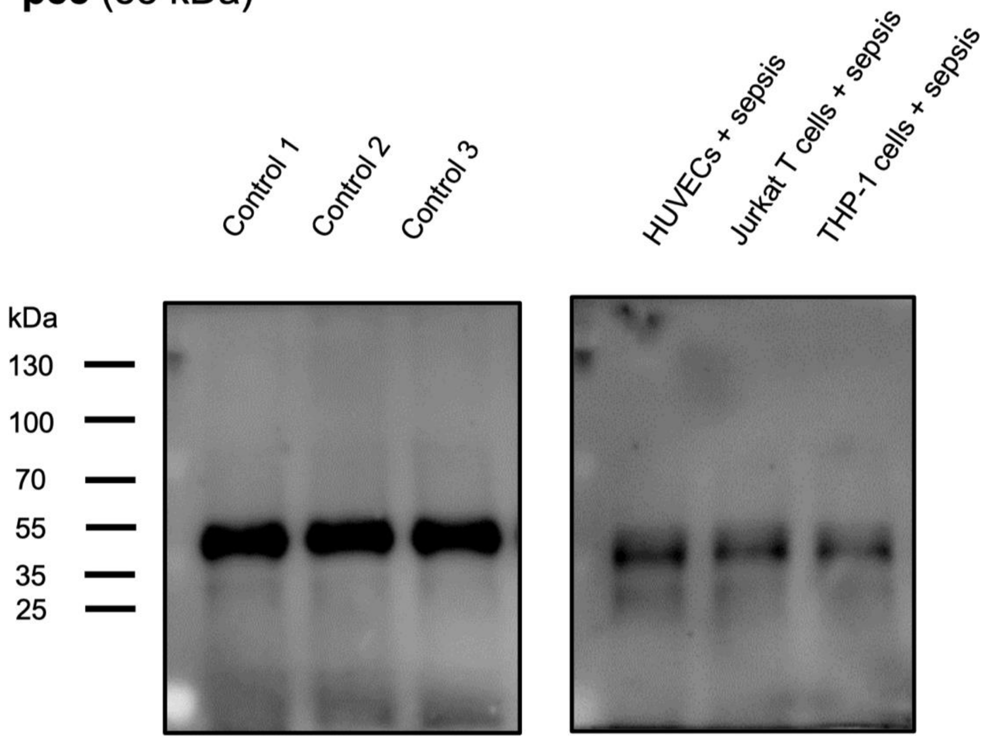

FasL (31 or 37 kDa)

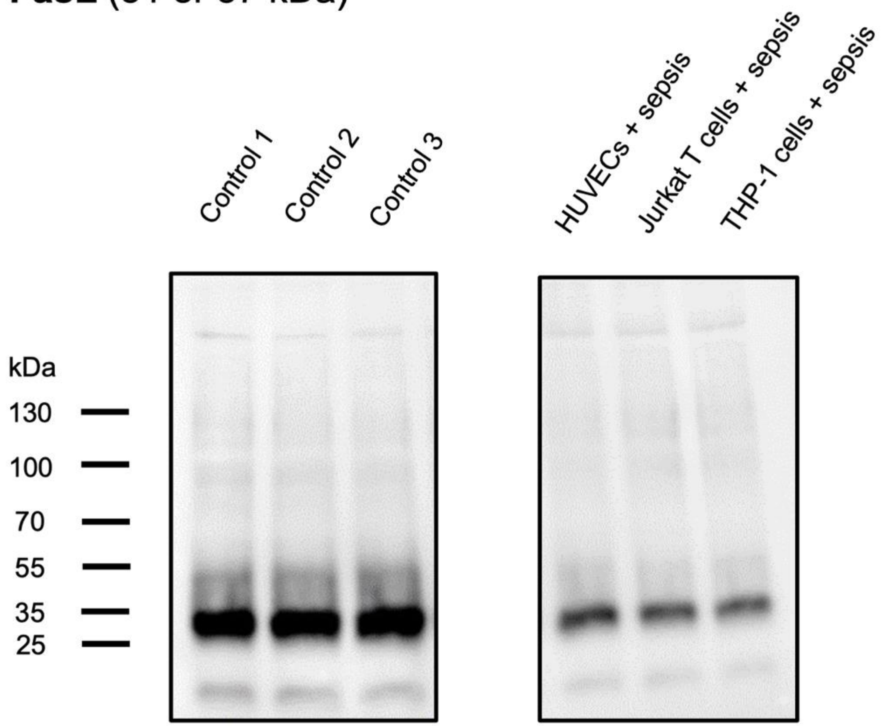

NLRP6 (99 kDa)

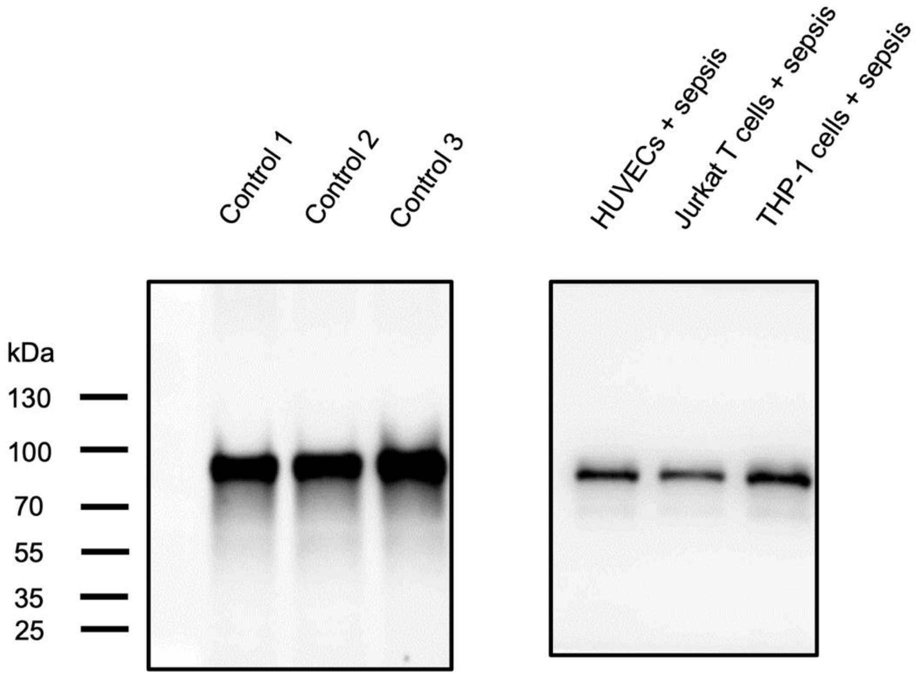

Supplement: Supplementary file 3 — Appendix S3. [file JCMM-29-e70582-s001.pdf]
